# Inverted All-Inorganic Nanorod-Based Light-Emitting Diodes via Electrophoretic Deposition

**DOI:** 10.1021/acsanm.4c03891

**Published:** 2024-10-07

**Authors:** Yongliang Zhang, Na Jia, Devika Laishram, Khizar Hussain Shah, Lin Lyu, Mei-Yan Gao, Pai Liu, Xiao Wei Sun, Tewfik Soulimane, Zhenhui Ma, Christophe Silien, Kevin M. Ryan, Ning Liu

**Affiliations:** † Department of Physics and Bernal Institute, University of Limerick, Limerick V94 T9PX, Ireland; ‡ Department of Chemical Sciences and Bernal Institute, University of Limerick, Limerick V94 T9PX, Ireland; § Institute of Nanoscience and Applications, and Department of Electrical and Electronic Engineering, Southern University of Science and Technology, Shenzhen, Guangdong 518055, China; ∥ Shenzhen Key Laboratory of Deep Subwavelength Scale Photonics, Southern University of Science and Technology, Shenzhen 518055, China; ⊥ Department of Physics, Beijing Technology and Business University, Beijing 100048, China

**Keywords:** nanorod-based light-emitting diodes, inverted architecture, electrophoretic deposition, vertically aligned nanorods, all-inorganic

## Abstract

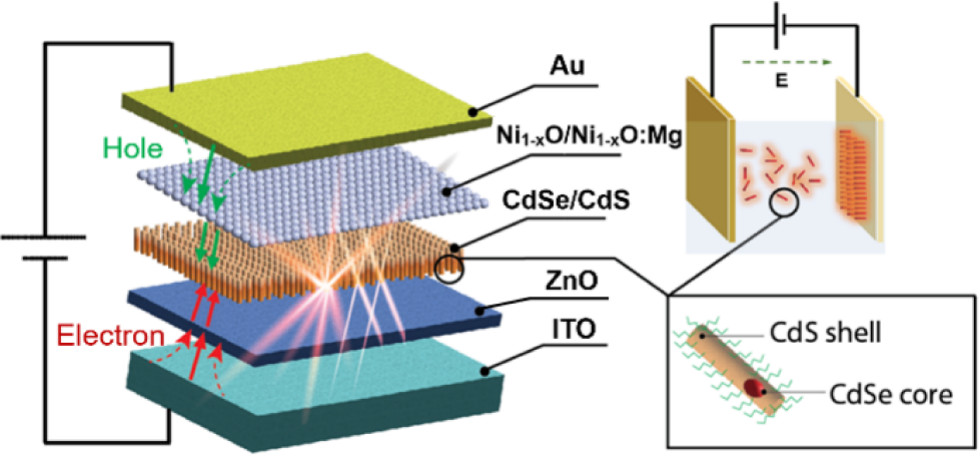

High performance and high stability in all-inorganic solution processed nanocrystal-based light-emitting diodes (LEDs) are highly attractive for large area devices compared to organic material-based LEDs. In this work, an inverted all-inorganic LED structure is designed to have an easy integration with thin-film transistors. Adopting robust inorganic materials such as Ni_1–*x*_O nanoparticle films as a hole transport layer (HTL) is beneficial for the performance of LED. Herein, we have optimized the HTL by introducing Mg into Ni_1–*x*_O to bridge the difference in energy offset between the nanorod emissive layer and the HTL, in addition to the advantages of low temperature solubility of Ni_1–*x*_O:Mg nanoparticles. Furthermore, CdSe/CdS-based nanorods via electrophoretic deposition (EPD) are amassed in a vertically aligned (VA-NR) fashion as an emissive layer to facilitate the carrier transportation. Fostering these approaches enabled an EQE of 1.2% of the fabricated device, establishing the viability for further development of efficient and highly stable nanocrystal-based LEDs.

## 1 Introduction

High efficiency, saturated colors, high stability, and long operational lifetimes are imperative for next-generation displays (e.g., metaverse, augmented reality, and naked-eye 3D displays). As a promising candidate that meets these requirements, nanocrystal-based light-emitting diodes (NC-LEDs) have become a growing area of research because of their attractive features, such as narrow emission bandwidths, stable optical performance, and suitability for high pixel density screen resolution.^1,2^ An inverted LED with a structure of ITO (cathode)/electron transport layer (ETL)/NC layer/hole transport layer (HTL)/metal anode is considered to be more advantageous than a standard one (ITO (anode)/HTL/NCs/ETL/metal cathode) from a display manufacturing aspect. Inverted devices can reduce the driving voltage of pixels and stabilize the devices, as the transparent bottom cathode of inverted NC-LEDs can be directly linked to the drain line of an n-type thin-film transistor (TFT).^3^ In addition, the inverted structure is well compatible with n-type active matrix TFTs.^4^

Significant progress has been made to improve the device efficiency, stability, and long operational lifetime of inverted NC-LEDs with an inorganic–organic hybrid structure, where NCs are sandwiched between an inorganic ETL and an organic HTL.^5,6^ In particular, economical and efficient solution-deposition routes, such as spin-coating and inkjet printing for organic HTLs,^2,7−9^ have been developed that can in principle replace vacuum deposition techniques to reduce costs. However, studies have shown that some organic materials can deteriorate the stability of NC-LEDs, e.g., polyethylene dioxythiophene: polystyrene (PEDOT: PSS) can trigger stability issues due to its acidic and hygroscopic properties, leading to long-term operating failure. This is a result of exciton-induced degradation of the organic HTL and the subsequent exciton quenching in the NC emitting layer.^10,11^ The formation of nonradiative recombination sites, and consequently, exciton-induced permanent material degradation, is another issue in hybrid inverted NC-LEDs, which can lead to a decrease in the luminescence quantum yield of NCs and subsequent device degradation.^10^

An inorganic HTL layer, on the other hand, offers high stability in oxygen-rich and humid environments, supports high injection currents, and meets the facile packaging requirements that are highly sought after for device fabrication.^12,13^ P-type metal oxides have been used as HTLs in conventional NC-LED structures according to their wide bandgap and switchable work function.^14−16^ One popular choice of p-type metal oxide is NiO. Pure, stoichiometric NiO is a good insulator, with a conductivity on the order of 10^–13^ S/cm at room temperature.^17^ On the contrary, nonstoichiometric Ni_1–*x*_O is a p-type semiconductor with a wide bandgap,^18^ whose properties are highly sensitive to preparation methods and postdeposition treatments.^19^ Solution-processed Ni_1–*x*_O has been used as an HTL in conventional NC-LED architecture due to its low cost, room temperature processing, simplicity,^20^ and suitable work function (WF), which provides good contact for hole injection and creates an energy barrier for electron blocking.^16^ However, poor mobility and strong interactions between NCs and the metal oxide HTL hinder device performance.^21^ Therefore, treatments such as Mg doping in the Ni_1–*x*_O HTL have been employed to modulate the WF and improve hole mobility in Ni_1–*x*_O.^22^ Another challenge in using solution-processed Ni_1–*x*_O as the HTL in an inverted structure is solvent erosion on the NC emission layer. NCs (e.g., quantum dots and nanoplates) are usually dispersed in common organic solvents, such as toluene, hexane, and chlorobenzene before deposition. They can be easily redissolved during the HTL coating process, leading to severe shorting and nonemissive recombination problems in NC-LEDs. As a result, solvent selection is critical for the fabrication of all-inorganic inverted NC-LEDs.

Highly luminescent CdSe/CdS core–shell NRs are excellent emissive materials because of their tunable emission wavelengths similar to QDs, a larger Stokes shift, faster radiative decay processes, linearly polarized emission, and slower bleaching kinetics than QDs.^23,24^ However, due to its geometric anisotropy, spin-coated NR films exhibit more voids compared to those of QD films. More layers of NRs therefore need to be deposited in order to decrease the leakage current,^25^ which leads to lower current density and shows no advantages over QD-based devices. To address the issue of low current density, we have shown that long native capping ligands (14-carbon chain) on the NRs can be replaced by shorter capping ligands (8-carbon chain) through ligand exchange, which leads to a 10-fold increase in injection current.^26^ In addition, our group recently demonstrated the application of vertically aligned NR films as an emission layer in a hybrid LED architecture, showing an improved performance compared to spin-coated NR films owing to the void-free film morphology and better charge transport along the long axis of the NRs.^25^

In this study, we demonstrate all-inorganic NR-LEDs in an inverted architecture for the first time. Only intrinsically robust inorganic materials are used in device fabrication. ZnO is used as the ETL, which is competent for highly efficient NC-LEDs and is confirmed to be a good choice as the electron transport material.^7,27^ Ni_1–*x*_O and Mg-doped Ni_1–*x*_O (Ni_1–*x*_O:Mg) nanoparticles, dispersed in a solvent mixture of ethanol and ethanolamine, are deposited over the emission layer by spin coating, which is easy to operate and is a low-cost method. Ethanol is orthogonal from the active material solvent (e.g., toluene and hexane). It is a mature and common solvent used in the conventional structure of NC-LEDs and does not affect the emissive layer. When these NRs are spin-coated onto ZnO, followed by the subsequent deposition of Ni_1–*x*_O or Ni_1–*x*_O:Mg NP layers, all-inorganic inverted NR-LEDs are successfully fabricated with a turn-on voltage as low as 3.7 V, an EQE of 0.15%, and a luminance of 3290 cd/m^2^ at 10 V for devices using the Ni_1–*x*_O:Mg NP layer as the HTL. Once the electrophoretic deposition technique (EPD) is applied to form a vertically aligned, closely packed NR emissive layer,^25,28^ the maximum EQE of the devices is further boosted to 1.2%.

## 2 Experimental Section

### CdSe/CdS Core–Shell Nanorods Synthesis

CdSe/CdS nanorods were synthesized based on the previously reported literature with some fine optimizations.^29^ First, the CdSe core (seeds) was synthesized as described in our previous work.^25^ The as synthesized CdSe core solution was then dispersed in toluene and then centrifuged at 10000 rpm for 10 min. Afterward, the precipitate was removed and isopropanol was added to the solution. The mixture was washed at 5000 rpm for 5 min, and the precipitate was finally dissolved in TOP. To synthesize the CdSe/CdS nanorods, similar steps were undertaken with the exception of injecting 2.4 g TOP. Detailed recipe can be found in our previous work.^25^

### Ni_1–*x*_O and Ni_1–*x*_O:Mg NP Synthesis

Ni_1–*x*_O nanoparticles were codeveloped with Shenzhen Planck Innovation Co Ltd. For the synthesis of Ni_1–*x*_O nanoparticles, Ni(NO_3_)_2_ • 6H_2_O (4.5 mmol) and dimethyl sulfoxide (30 mL) were first mixed in a round-bottom three-neck flask. Then, a 10 M NaOH solution was added to the mixture drop by drop until the pH reached 10. The obtained solution showed a turbid green color and was maintained at 160 °C in air for 3 h. The solution was then centrifuged and washed with deionized water two times. Finally, the as-synthesized nanoparticles were dispersed in ethanol for further use. Ni_1–*x*_O:Mg nanoparticles were prepared similarly by adding a mixture of Ni(NO_3_)_2_• 6H_2_O and Mg(NO_3_)_2_• 6H_2_O in the required stoichiometric amounts, in the current case, specifically a 99:1 ratio. The rest of the steps were the same as those in the Ni_1–*x*_O nanoparticle preparation method.

### Characterizations

A FEI Helios G4 CX FIB/SEM system was used to acquire the surface and cross-sectional SEM images at 5 kV. TEM images of CdSe/CdS NRs were obtained on a JEOL JEM-1011 microscope at a 100 kV accelerating voltage. TEM images of the cross sections of the Ni_1–*x*_O and Ni_1–*x*_O:Mg films on Si were obtained by Thermofisher Talos TEM F200i at 200 kV accelerating voltage. Diluted NR colloidal suspensions were drop-cast onto 200-mesh carbon-coated copper grids for TEM inspection. X-ray photoelectron spectroscopy (XPS) was conducted using a Kratos Axis-Ultra spectrometer with a monochromatic Al K_α_ source operating at 15 kV and 8 mA. An X-ray diffractometer (D8 Advance, BRUKER) in the diffraction angle range from 20° to 70° with Cu K_α_ radiation was used to determine the crystal phase composition and structure of the nanocrystals.

### LED Device Fabrication and Testing

Patterned ITO glasses were used as substrates for the NR-LEDs. These ITO glasses were first cleaned by sonication in acetone and isopropanol sequentially. The ITO substrates were further treated with oxygen plasma for 5 min at 15 W prior to ZnO deposition. The ETL ZnO layer was deposited on the cleaned ITO substrates by atomic layer deposition (ALD, Anric AT-400). Diethylzinc (Sigma-Aldrich) and water were used as precursors. During the ALD growth, the chamber temperature was kept at 150 °C, and the Ar flow was maintained at 200 mTorr. 25 nm ZnO was obtained after 260 cycles. For the emission layer prepared by EPD, the NR layer was deposited at 300 V for 180 s from a NR solution with a concentration of 0.8 mg/mL.^25^ Ni_1–*x*_O/Ni_1–*x*_O:Mg nanoparticles were dissolved in a solvent mixture of ethanol and ethanolamine at 20 mg/mL and spin-coated as the HTL at 3000 rpm for 30 s, followed by annealing at 120 °C for 10 min. Finally, Au was deposited by thermal evaporation to a thickness of 40 nm. The current–voltage–luminance characteristics were measured using a Keithley 2410 source unit and an integrating sphere coupled to a photodiode (Thorlabs SM1PD1A). The output of the Si photodiode was connected to a current amplifier (Thorlabs PDA 200C). The EQE was calculated as the ratio of the photon flux and the driving current of the device. The EL spectra of the devices were obtained using an Ocean Optics HR4000+ spectrometer.

## 3 Results and Discussion

**Figure 1:**
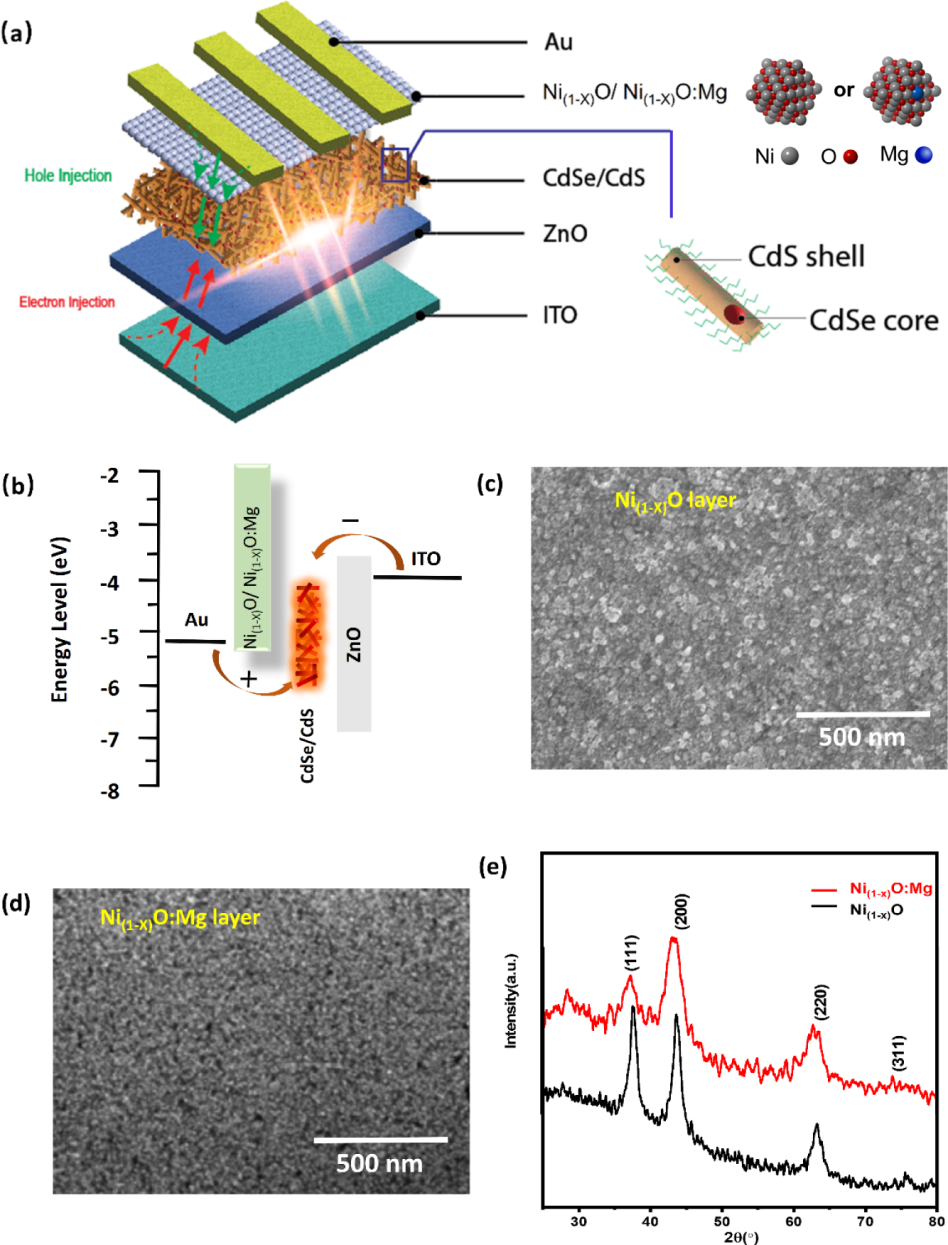
(a) Device structure of the multilayer LED made by spin coating. (b) Energy level diagram for the function layers in the device. (c) and (d) SEM image of Ni_1–*x*_O and Ni_1–*x*_O:Mg nanoparticles by spin coating. (e) XRD pattern of Ni_1–*x*_O and Ni_1–*x*_O:Mg films.

The structure of our device architecture with a spin-coated emissive layer and the corresponding energy level diagram of the NR-LED device are shown in Figure 1a,b, respectively. ITO on glass substrates is used as the negative electrode. ZnO is deposited as the ETL using atomic layer deposition, which offers a smooth morphology for the ZnO film, essential for the later deposition of the NR layer and for improving electron transport performance. The CdSe/CdS NR was spin-coated as the emission layer (Figure S1a). Next, Ni_1–*x*_O/Ni_1–*x*_O:Mg nanoparticles were spin-coated as HTLs and Au was deposited by thermal evaporation as the anode. The energy levels for different layers were obtained from previous works.^30−32^

The morphological analysis of the as-synthesized materials was characterized by using scanning electron microscopy (SEM) and transmission electron microscopy (TEM). The TEM image of CdSe/CdS NRs is shown in Figure S1b. The NRs were observed to have a length of 32 ± 3 nm and a width of 4.8 ± 0.2 nm. The aspect ratio was around 7:1–6:1, which was suitable for further use in the fabrication by EPD. X-ray diffraction (XRD) analysis was performed to investigate the crystal phase and the average particle size of the CdSe/CdS core–shell nanorods. As shown in Figure S1c, the wurtzite crystalline phase with a 4.8 nm particle diameter of the CdSe/CdS core–shell NRs was obtained using XRD analysis. All diffraction peaks were very consistent with the standard wurtzite CdS crystal XRD pattern (JCPDF of No. 41-1049). In addition, the narrow peak widths indicate the crystalline nature of the synthesized materials. The peak at 26° in the diffraction pattern corresponds to the reflections of the (002) crystal plane. This indicates that the nanorods have a bipolar disposition owing to the noncentrosymmetric charge distribution in wurtzite crystals along the *c*-axis. This coupled charge distribution is advantageous during device fabrication in EPD. The CdSe core with CdS shell shows high photoluminescence (PL) intensity at the wavelength of 612 nm and the PL quantum yield (QY) in film is 55% (Figure S1d).

Figure 1c shows the surface morphology of the Ni_1–*x*_O film by spin coating on the Si substrate. The uniform coating with a particle size of around 20–30 nm can be observed in the SEM image. In our case, the prepared Ni_1–*x*_O nanoparticles were dispersed in the mixture solvent of ethanol and ethanolamine with a volume ratio of 10:1, and then the Ni_1–*x*_O layer was obtained by spin coating with a concentration of 20 mg/mL at 3000 rpm for 30 s. Uniformly distributed Ni_1–*x*_O:Mg particles (around 5 nm in length) with smooth surface morphology formed by spin coating on the Si substrate are observed in the SEM image as shown in Figure 1d. It can be deduced that the quality of the film is enhanced in Mg-doped Ni_1–*x*_O. TEM images on the cross sections of films of Ni_1–*x*_O and Ni_1–*x*_O:Mg films on Si are given in Figure S2, showing a denser film formation in the Ni_1–*x*_O:Mg case.^33^

The XRD pattern for Ni_1–*x*_O in Figure 1e shows diffraction peaks at 37.26°, 43.31°, and 62.98°, which can be assigned to (111), (200), and (220) planes, well matched with the bunsenite cubic structural NiO in the space group Fm3m (NaCl-type) corresponding to JCPDF card no. 75-0197. The crystal structure of the spin-coated Ni_1–*x*_O:Mg films observed from the X-ray diffraction pattern exhibited distinct diffraction peaks at 37.42°, 43.40°, and 63.19° degrees corresponding to (111), (200), and (220) planes as can be seen in Figure 1e. It should be noted that the peaks around 29° can be attributed to the small amount of unfinished Ni(NO_3_)_2_•(6)H_2_O/Mg(NO_3_)_2_•(6)H_2_O.^34^

The average crystallite sizes of nickel oxide nanoparticles were calculated by the Debye–Scherer equation. There is a 0.05° right shift in the XRD diffraction, corresponding to the crystallite size of Ni_1–*x*_O nanoparticles decreased from 20 to 5 nm in the Ni_1–*x*_O:Mg (Figure 1c,d) nanoparticles. With the Mg doping, the full-width half-maximum (FWHM) of diffraction peaks was found to be broadened with the intensity of the diffraction peaks decreased, indicating that a certain amount of Mg doping can inhibit the growth of Ni_1–*x*_O NCs.

**Figure 2:**
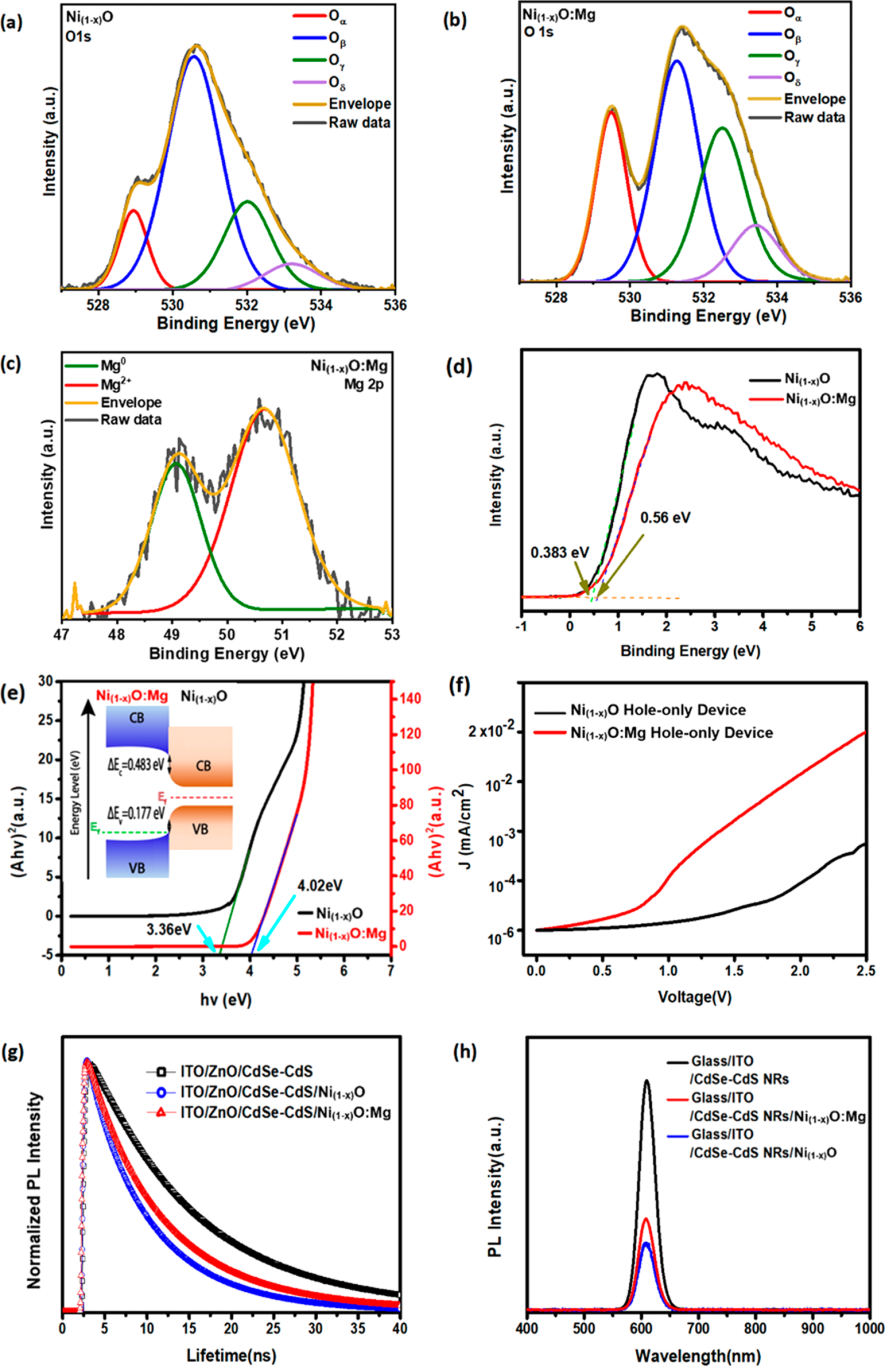
(a)–(c) XPS spectra of Ni_1–*x*_O and Ni_1–*x*_O:Mg films. (d) XPS valence band spectrum for Ni_1–*x*_O and Ni_1–*x*_O:Mg. (e) Band gaps for Ni_1–*x*_O and Ni_1–*x*_O:Mg (Inset image: Band diagrams of Ni_1–*x*_O and Ni_1–*x*_O:Mg). (f) J–V characteristics of hole-only devices with the structure of ITO/HTL/NRs/HTL/Au. (g) TRPL of NRs deposited with different HTLs. (h) PL of NRs on different HTLs.

To understand the nature of chemical behavior in the films and to investigate the changes brought about by Mg doping, XPS measurements were carried out (Figure 2a–c). A detailed table regarding the peak information is given in Table 1. The Ni peaks were deconvoluted by following a Gaussian–Lorentzian function. In the case of both Ni_1–*x*_O and Ni_1–*x*_O:Mg films, the Ni 2p_3/2_ peaks were observed to contain three main peaks and corresponding three satellite peaks. As shown in Figure S3a, the peaks for the Ni_1–*x*_O film were noted at 853.9, 855.6, and 857.3 eV, which can be assigned to NiO (Ni^2+^), Ni(OH)_2_ (Ni^2+^)/NiOOH (Ni^3+^), and Ni intersite (Ni^3+^) and satellite peaks at 860.4, 862.8, and 865.8 eV, which is in accordance with that reported in the literature.^35−37^ The high-resolution XPS spectrum of Ni_1–*x*_O:Mg shows peaks at 854.3, 855.8, and 857.7 eV corresponding to NiO, Ni(OH)_2_/NiOOH, and Ni intersite, respectively, with corresponding satellite peaks at 860.9, 863.6, and 866.5 eV (Figure S3b). The Ni^2+^ peak represents stoichiometric NiO, which is a Mott–Hubbard insulator, while the Ni^3+^ peak illustrates off-stoichiometric NiO, representing Ni^2+^ vacancies in the NiO film that induces the formation of Ni^3+^. Therefore, it can be inferred that a higher Ni^3+^-related peak intensity in Ni_1–*x*_O:Mg compared to Ni_1–*x*_O signifies more Ni^2+^ vacancies introduced throughout the film, a resultant of the introduction of Mg in Ni_1–*x*_O.^38^ Consequently, a higher Ni^3+^/Ni^2+^ ratio in Ni_1–*x*_O:Mg films than that in Ni_1–*x*_O films can be derived from Table 1. Furthermore, Ni^3+^ being an acceptor can contribute to the conductivity in the Ni_1–*x*_O:Mg films to boost the device performance when used as an HTL in the NR-LED.

**Table 1:** Summary of the XPS Analysis Results

		Ni_1–*x*_O		Ni_1–*x*_O:Mg	
		Conc. %	Position	Conc. %	Position
**O 1s**	O_α_ (NiO)	5.9	529	9.9	529.5
	O_β_ (Ni(OH)_2_)	32.5	530.6	18.1	531.3
	O_γ_ (NiOOH)	10.6	532.0	12.8	532.5
	O_γ_ (H_2_O)	3.7	533.2	4.6	533.4
**Ni 2p_3/2_**	Ni^2+^(NiO)	3.8	853.9	3.7	854.3
	Ni^2+^ (Ni(OH)_2_)/Ni^3+^ (NiOOH)	4.3	855.6	5.1	855.8
	Ni^3+^ (Intersite)	1.5	857.3	2.2	857.3

The O 1s peak in Figure 2a is deconvoluted into four peaks using a Gaussian–Lorentzian distribution, centered at approximately 529 eV (O_α_), 530.6 eV (O_β_), 532.0 eV (O_γ_), and 533.2 eV (O_δ_). These peaks correspond to O 1s in NiO, Ni(OH)_2_, NiOOH, and H_2_O, which can be attributed to lattice oxygen, surface-adsorbed oxygen species, surface-adsorbed hydroxyl oxygen and moisture present. The O 1s spectrum for Ni_1–*x*_O:Mg (Figure 2b) exhibited peaks at the same binding energy levels as Ni_1–*x*_O with a difference of ±0.5 eV. For both Ni_1–*x*_O and Ni_1–*x*_O:Mg, the relative ratios of surface oxygen present were calculated using the formula O_β_/ (O_α_ + O_β_ + O_γ_), resulting in values of 61.67% and 39.86%, respectively. This indicates that defects created in the Ni_1–*x*_O:Mg film might see enhanced passivation as a consequence of Ni–Mg bond formation.^39^ The high-resolution Mg 2p spectra are given in Figure 2c with two peaks fitted at 49.1 and 50.7 eV using a similar Gaussian–Lorentzian function fitting and can be assigned to the metallic Mg^0^ and Mg^2+^, respectively, with a Mg^0^/Mg^2+^ concentration ratio of 0.54. More information can be found in Table S1.

To get the band position of Ni_1–*x*_O and Ni_1–*x*_O:Mg, XPS valence band (VB) spectra was employed to investigate the VB energy levels (Figure 2d). The maximum energy edges of VB density of states for Ni_1–*x*_O and Ni_1–*x*_O:Mg were approximately at 0.38 ± 0.02 and 0.56 ± 0.02 eV, respectively. The VB of Ni_1–*x*_O shifted down by a value of 0.18 ± 0.02 eV via Mg doping, consistent with the literature.^40^ UV–vis was conducted to further estimate the band gap values of the Ni_1–*x*_O and Ni_1–*x*_O:Mg samples. There was no obvious change in color of the Ni_1–*x*_O and Ni_1–*x*_O:Mg solutions while their light absorption spectra showed a blue shift of the bandgap absorption shoulders from 310 nm (Ni_1–*x*_O) to 287 nm (Ni_1–*x*_O:Mg) as shown in Figure S4, indicating an increased bandgap for Ni_1–*x*_O:Mg NPs. The band gaps of Ni_1–*x*_O and Ni_1–*x*_O:Mg were estimated on the basis of the Kubelka–Munk (KM) method.^41^ The estimated band gaps for Ni_1–*x*_O and Ni_1–*x*_O:Mg were 3.36 and 4.02 eV, as shown in Figure 2e, respectively. The inset image in Figure 2e depicts the deduced band diagram for Ni_1–*x*_O and Ni_1–*x*_O:Mg. There is a Δ*E*_v_ difference of 0.18 eV and a Δ*E*_c_ difference of 0.438 eV for Ni_1–*x*_O:Mg compared to Ni_1–*x*_O, likely due to the creation of more holes from Mg doping, which pins the Fermi level closer to the top of VB.^42^ These results show that the band gap of Ni_1–*x*_O increases significantly with Mg doping (0.66 eV). Thus, the charge transfer efficiency is greatly improved after doping, which is attributed to the decrease in the barrier between the HTL and the active layer, along with higher electron blocking.

To verify the enhancement in hole transport, hole-only devices are characterized by induced currents. These devices are fabricated with the structure ITO/HTLs (∼40 nm)/NRs (∼30 nm)/HTLs (∼40 nm)/Au (∼100 nm) as shown in Figure S5. The J–V characteristics of the hole-dominant devices are compared in Figure 2f. The Ni_1–*x*_O:Mg device exhibits a higher current density owing to the reduced hole injection barrier and the increased concentration of Ni^3+^ in the Ni_1–*x*_O:Mg layer.

The difference in luminescence behavior was further studied by time-resolved PL. Figure 2g shows the exciton lifetime of NRs on ZnO/ITO/Glass (17.2 ± 0.8 ns) with different HTLs. The average lifetime of sample ITO/ZnO/NRs/Ni_1–*x*_O is 12.7 ± 0.7 ns, while the increased exciton lifetime (15.2 ns ±0.8) is observed on the sample with Ni_1–*x*_O:Mg film, which may be attributed to an effective defect passivation by Mg-doping in Ni_1–*x*_O nanoparticles. Meanwhile, the corresponding fluorescence property of NRs with different HTLs is investigated by photoluminescence (PL) spectra (Figure 2h). Even though the PL intensity of ITO/ZnO/NRs/HTL decreases from that of ITO/ZnO/NRs, the sample with Ni_1–*x*_O:Mg shows less intensity quenching compared to Ni_1–*x*_O.

**Figure 3:**
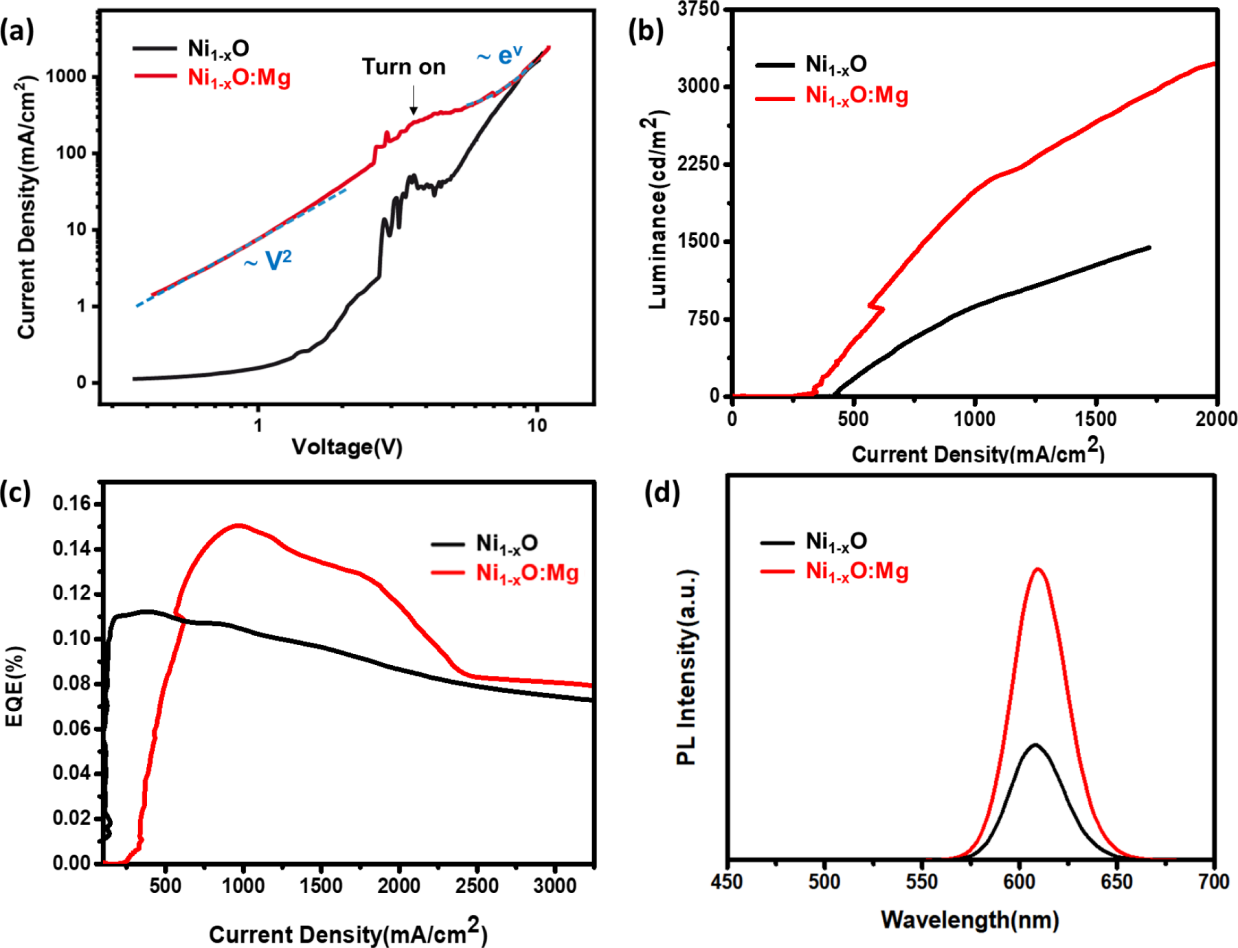
Characteristics of LEDs with different HTLs. (a) Current density–voltage curves, (b) luminance–current density, (c) EQE – current density and (d) the EL spectra of LED with Ni_1–*x*_O and Ni_1–*x*_O:Mg LEDs.

The performance of the NR-LEDs was characterized by analyzing various parameters, such as current density, luminance, and voltage characteristics (Figure 3a–c). For the device with Ni_1–*x*_O:Mg as the HTL, at low injection current (forward bias <2 V), the current density increases proportionally to V^2^, indicating that the device operates in the space-charge limited region, most likely due to the medium dopant levels of the HTL.^43,44^ Above 2 V, the onset of electron–hole recombination in the active layer allows the current density to increase faster than the square of the applied bias. At relatively high injection currents (forward bias >7 V), other processes such as Auger recombination and carrier leakage occur, which is reflected in the J–V curve as the current density increases exponentially with the applied voltage. For the device with a Ni_1–*x*_O NP layer as the HTL, a lower injection current is observed below 1.5 V due to the higher energy barrier between the Ni_1–*x*_O HTL and the CdSe/CdS NRs. At higher injection currents (forward bias >8 V), a similar exponential increase in current density vs applied voltage is observed, suggesting that the same processes dominating the J–V characteristic ofthe Ni_1–*x*_O:Mg device are also present. Moreover, the compactness of the HTL film plays an important role in the charge transport capabilities of the semiconductor films. It was observed that denser Ni_1–*x*_O:Mg film is favorable for enhancing hole carrier mobility and decreasing the device current leakage.^45^

The turn-on voltages of the NR-LEDs are 3.7 and 4.2 V for Ni_1–*x*_O:Mg and Ni_1–*x*_O devices, respectively, providing further evidence of the reduced hole injection barrier from Ni_1–*x*_O:Mg to the CdSe/CdS emission layer compared to that of the Ni_1–*x*_O devices. In addition, the driving voltage of the devices with the Ni_1–*x*_O:Mg layer is reduced substantially at high current density compared to the Ni_1–*x*_O device. After doping of Ni_1–*x*_O nanoparticles by Mg, the peak brightness and maximum EQE of the device are further improved to 3290 cd/cm^2^ (Figure 3b) and 0.15%, respectively (Figure 3c). The Ni_1–*x*_O:Mg LED shows a higher EL than the Ni_1–*x*_O one as shown in Figure 3d.

**Figure 4:**
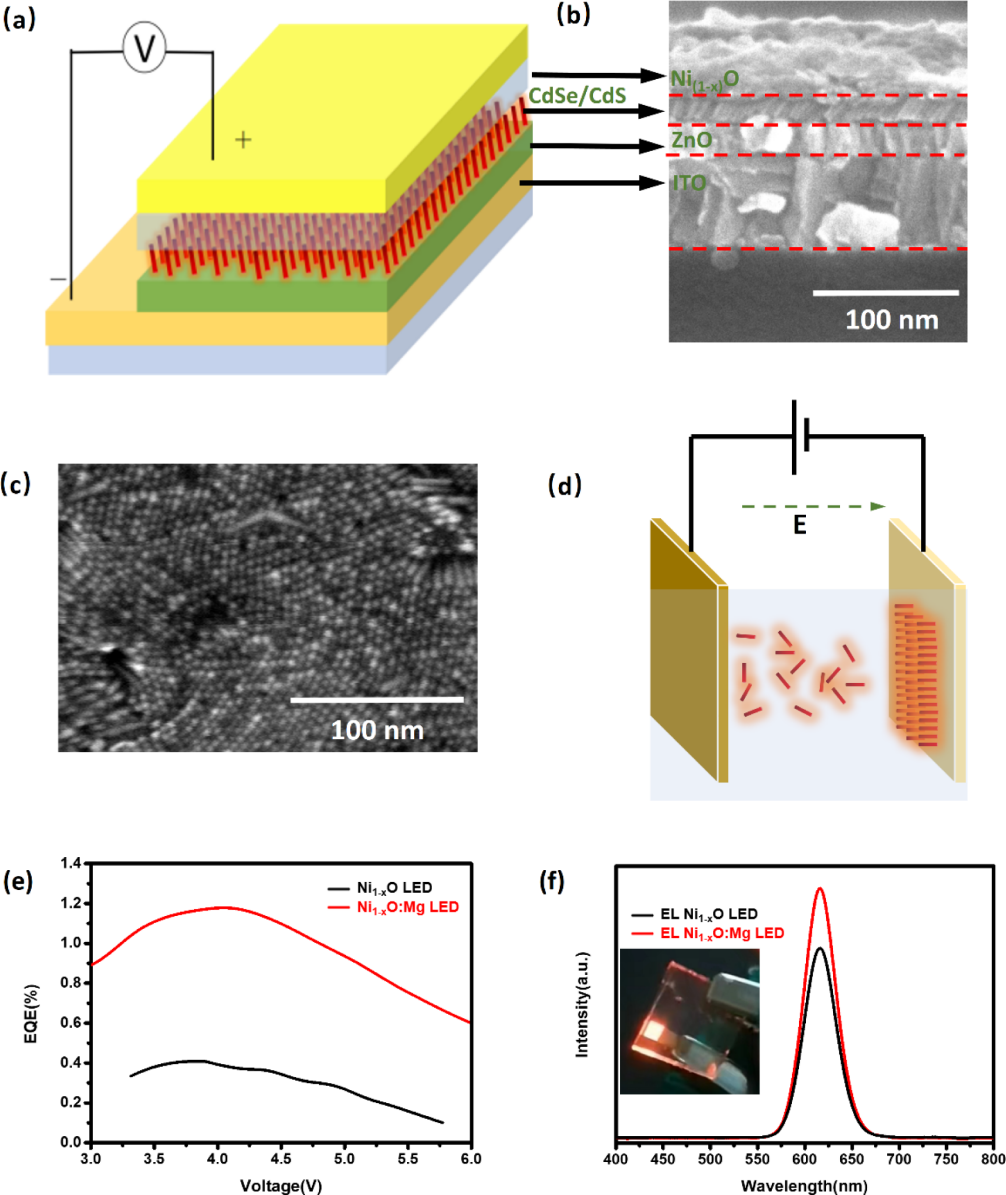
Device structure of the multilayer LED (a), cross-section image of a LED device by EPD (b), top view image of NRs film by EPD (c), and schematic diagram of EPD with CdSe/CdS NRs (d). Characteristics of the LEDs with Ni_1–*x*_O and Ni_1–*x*_O:Mg HTLs, EQE – voltage curves (e) and EL spectra of LED with Ni_1–*x*_O and Ni_1–*x*_O:Mg LEDs (f). The inset shows an NR-LED by EPD operating at 5 V.

High performance NC-LED devices are achieved with ordered arrays of NCs, which can minimize the electrical resistance and efficiently enhance the confinement of excitons.^46^ The features of the NC active layer namely, surface morphology, interface traps, and energy alignment, highly influence the performance of NC-LEDs and the quenching process occurring at the interfaces of CTLs/NCs, which decreased the efficiency of previously reported all-inorganic devices.^47,48^ With the aim to enhance the stability and performance of the LED, based on the optimized HTL conditions, uniform and vertically aligned (VA) CdSe/CdS NR layer is obtained by EPD as the emission layer.^25^ A schematic structure of the vertically aligned NR-LED, together with a typical cross-section view SEM image, are shown in Figure 4a,b. During the EPD, the charged NRs are accelerated by an applied voltage, in our case, around 300 V. The NRs carry relatively large kinetic energy (a few tens of eV) as they arrive at the substrate of opposite polarity. This ensures that the NRs have good contact with the substrate surface and among themselves, similar to the sputtered films. The NR-LED was fabricated in an inverted structure similar to the previous ones (spin coating) with the only difference wherein the emission layer was deposited by EPD with vertically aligned NRs as shown in Figure 4c. The device substrate (Glass/ITO/ZnO) is mounted to the cathode, while an ITO-glass serves as the anode. Both are connected to a high-voltage power supply. The EPD occurs in the CdSe/CdS nanorod solution under an electric field, resulting in the formation of a vertically aligned CdSe/CdS nanrod array on the device substrate.^25,28^ As shown in Figure 4b, each functional layer does not mix with the other layers. They are spatially separated. As can be observed, this device has a monolayer CdSe/CdS with a thickness of approximately 30 nm. The CdSe/CdS NRs were found to have vertical alignment and exhibit good film quality.

The Ni_1–*x*_O:Mg-based vertically aligned (VA) NR-LED slightly decreased the turn-on voltage from 3.0 to 2.7 V and increased the current density compared with the Ni_1–*x*_O device, as shown in the device performance characteristics in Figure S6a,b. The maximum EQE of the vertically aligned NR-LED was enhanced to 1.2% (Figure 4e), which is approximately an 8-fold enhancement compared with the spin-coated device. In addition, the FWHMs of 32 nm with the vertically aligned NR-LED were narrower, as shown by the EL spectra in Figure 4f. The improvement in the EQE of the EPD devices is attributed to better charge transport and reduced tiny voids that may exist in the film compared to a film formed by the simple spin-coating technique. The charge transport is improved from the perspective that fewer nanoparticle layers are needed for the same thickness of the active film so that the electron–hole recombination process within the same nanocrystal can happen more effectively.^25^

## 4 Conclusions

In conclusion, high-performance inverted all-inorganic LEDs with VA-NRs as the emission layer have been constructed. A peak EQE of 0.15% and a maximum luminance of 3290 cd/m^2^ were achieved, which can be attributed to the Ni_1–*x*_O:Mg_*x*_ particles lowering the hole injection barrier and, in turn, improving hole injection efficiency. Additionally, the performance of the inverted all-inorganic NR-LEDs was improved by the CdSe/CdS NR layer with a vertically aligned array and an enhancement in the EQE to 1.2% was obtained for VA-NR-LED. This improvement is attributed to the densely packed void-free film morphology of the active layer by EPD.^25^ It is noted that the EQE of our devices compared to the state-of-the-art QD-LEDs (usually with an organic–inorganic hybrid structure) is still relatively low (see Table S2). The main reasons for the lower EQE of all-inorganic NR-LEDs are attributed to (1) relatively large energy barrier between the NR emissive layer and the HTL and (2) unpassivated surface states at metal-oxide HTL, which is adjacent to the NR emissive layer. In this work, we demonstrated that lowering the hole injection barrier can improve the overall current density of the device. Further efficiency improvement can be achieved by ligand exchange in the nanorods and the treatment in the HTLs to passivate the surface states of the HTLs.^26^ We highlight the proposed strategy of a closely packed VA-NR film and demonstrate the promising future of the EPD film, for example, in applications such as polarized emission and electrically pumped NR lasers, where high current injection with a much thicker emissive layer is essential. We believe this provides a possible pathway for creating array films and could be generally applied to other film-based optoelectronic devices.

## Supporting Information Available

SEM image, TEM images, XRD and PL of CdSe/CdS core–shell NRs, TEM cross-sectional images of Ni_1–*x*_O and Ni_1–*x*_O:Mg films on Si, XPS of Ni 2p from Ni_1–*x*_O and Ni_1–*x*_O:Mg films, absorption spectra of Ni_1–*x*_O and Ni_1–*x*)_O:Mg NPs, the structure diagram of the hole-only devices, current density–voltage and luminance–voltage curves of EPD-LEDs with Ni_1–*x*_O (a) and Ni_1–*x*_O:Mg as HTLs, a summary table of the XPS analysis results of Mg 2p state of the Ni_1–*x*_O:Mg NPs, and a table comparing the performance parameters of NR-LEDs reported in recent years (PDF)
