# Supplementary material for: Inverted All-Inorganic Nanorod-Based Light-Emitting Diodes via Electrophoretic Deposition
Source: ACS Appl Nano Mater. 2024 Oct 7;7(20):23617–26. doi: 10.1021/acsanm.4c03891 (PMC11519866; doi:10.1021/acsanm.4c03891)
Supplement: Supplementary file 1 — an4c03891_si_001.pdf [file an4c03891_si_001.pdf]

Supporting Information

Inverted All-Inorganic Nanorod-Based Light Emitting Diodes Via Electrophoretic Deposition

Yongliang Zhang[1], Na Jia[2], Devika Laishram[1], Khizar Hussain Shah[1], Lin Lyu[1], Mei-Yan Gao[2], Pai Liu[3,4*], Xiao Wei Sun[3], Tewfik Soulimane[2], Zhenhui Ma[5], Christophe Silien[1], Kevin M. Ryan[2], Ning Liu[1,*]

[1]*Department of Physics and Bernal Institute, University of Limerick, V94 T9PX, Ireland*
[2]*Department of Chemical Sciences and Bernal Institute, University of Limerick, V94 T9PX, Ireland*
[3]*Institute of Nanoscience and Applications, and Department of Electrical and Electronic Engineering, Southern University of Science and Technology, Nanshan, Shenzhen, Guangdong, 518055, China*
[4]*Shenzhen Key Laboratory of Deep Subwavelength Scale Photonics, Southern University of Science and Technology, Shenzhen 518055, China*
[5]*Department of Physics, Beijing Technology and Business University, Beijing, 100048, China*
Emails: liup7@sustech.edu.cn, Ning.Liu@ul.ie

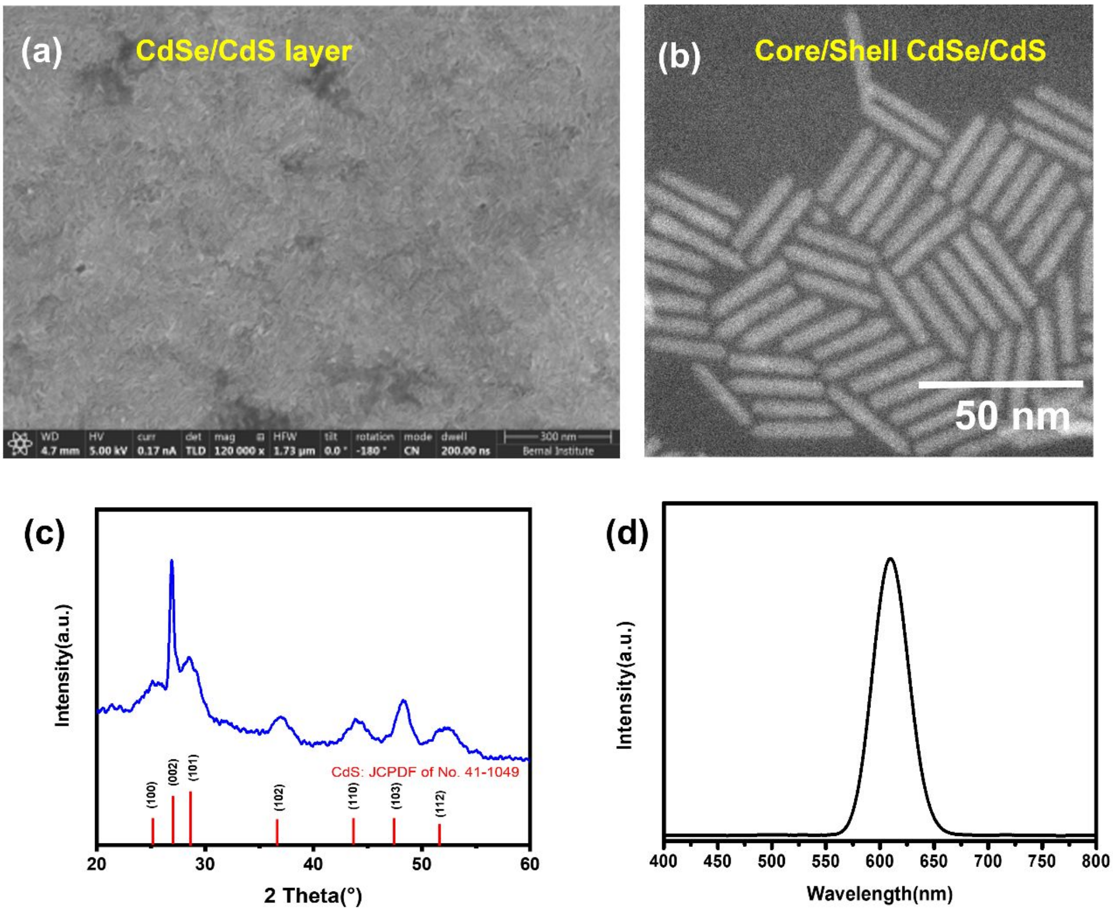

Figure S1 (a) The SEM image of CdSe/CdS nanorods layer made by spin coating. (b) TEM image of CdSe/CdS NRs (average length = 32nm, width = 4.8 nm). (c) X-Ray diffraction peak of the CdSe/CdS nanorods (d) PL spectra of the CdSe/CdS NRs

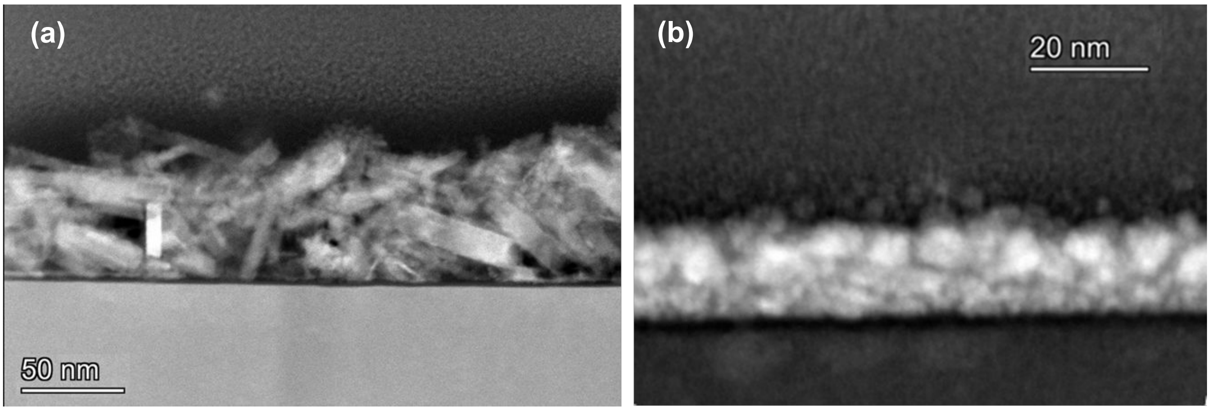

Figure S2: TEM images of the cross sections of $Ni_{(1-x)}O$ film on Si (a) and $Ni_{(1-x)}O$:Mg film on Si (b).

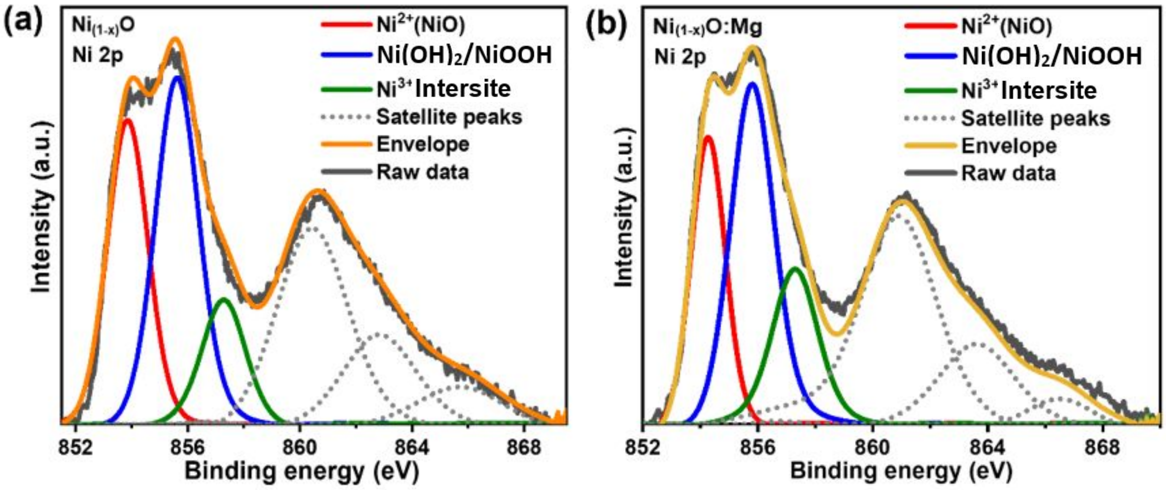

Figure S3: High resolution XPS of Ni 2p.

Table S1: Quantification from high resolution spectra of Mg 2p

Name	*Position* (eV)		*FWHM* (eV)		*Area*		*% Conc.*	
	$Ni_{(1-x)}O$	*$Ni_{(1-x)}O$:Mg*	*$Ni_{(1-x)}O$*	*$Ni_{(1-x)}O$:Mg*	*$Ni_{(1-x)}O$*	*$Ni_{(1-x)}O$:Mg*	*$Ni_{(1-x)}O$*	*$Ni_{(1-x)}O$:Mg*
Mg 2p (Mg^0)	-	49.1	-	1.1	-	311	-	1.3
Mg 2p (Mg^{2+})	-	50.7	-	1.4	-	558	-	2.4

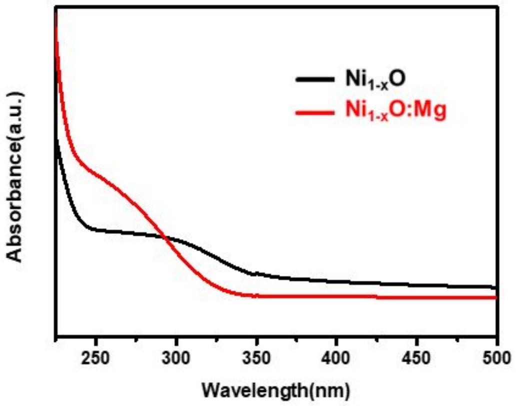

Figure S4 UV-Vis diffuse absorption spectra of $Ni_{(1-x)}O$ and $Ni_{(1-x)}O$:Mg

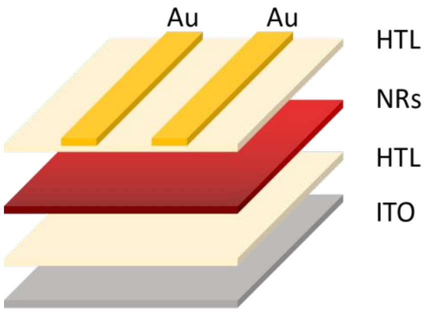

Figure S5 The structure diagram of the Hole-only device

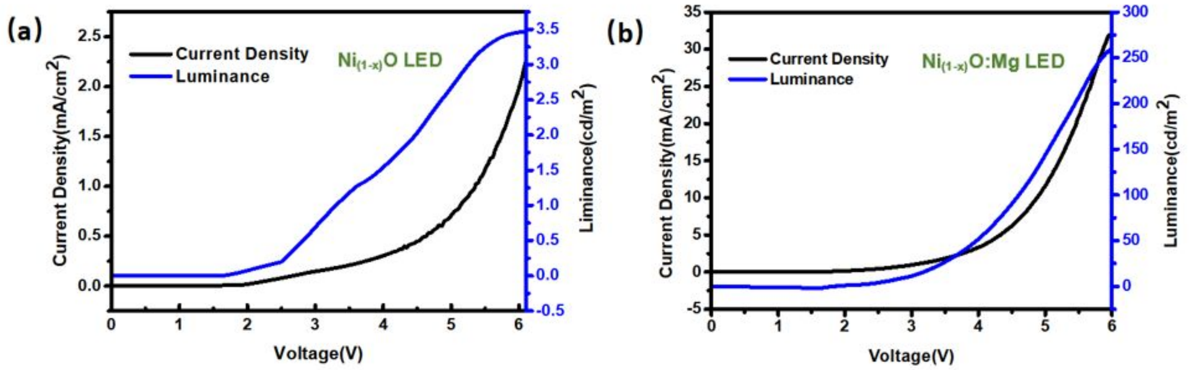

Figure S6 Characteristics of the LEDs with $Ni_{(1-x)}O$ (a) and $Ni_{(1-x)}O$:Mg HTLs (b), current density−voltage curves and luminance−voltage curves.

Table S2. Comparison of performance parameters of NR-LEDs reported in recent years

Nanorod	PLQY in film (%)	Max Luminance (cd/m^2)	EQE (%)	Method	Structure	Ref
CdSe/CdS/CdS	72	104000	15.7	Spin coating	ITO/PEDOT:PSS/TFB/NRs/PMMA/ZnO/Al	1
CdSe/CdZnS/ZnS	78	12000	21.5	Spin coating	ITO/PEDOT:PSS/poly-TPD/NRs/ZnMgO/Al	2
CdSe/CdS	40–50	8500	5.4	Spin coating	ITO/PEDOT:PSS/poly-TPD/PVK/NR/ZnO NPs/Al	3
CdSe/CdZnSe/CdZnS/ZnS	NA	160341	22	Spin coating	ITO/PEDOT:PSS/TFB/NRs/ZnO/Al	4
CdSe/CdS	44	3960	7	Spin coating	ITO/PEDOT:PSS/PVK/QRs/PMMA/ZnO/LiF/Al	5
CdS/CdSe-ZnSe	NA	7600	12.5	Spin coating	ITO/PEDOT:PSS/TFB/NRs/ZnO/Al	6
CdSe/CdS	NA	4320	6.3	EPD	ITO/ZnO/NRs/PVK/TFB/Au	7
CdSe/CdS	55	3290	1.2	EPD	ITO/ZnO/NRs/Mg-NiO/Au (Inverted All-Inorganic LED)	This work

1. Zhang, Yanbin, et al. "High-efficiency CdSe/CdS nanorod–based red light–emitting diodes." Optics express 27 (2019): 7935-7944.
2. Zeng, Yicheng, et al. "High-Efficiency and Stable Colloidal One-Dimensional Core/Shell Nanorod Light-Emitting Diodes." Nano Letters 24 (2024): 5647–5655.
3. Rastogi, Prachi, et al. "Enhancing the performance of CdSe/CdS dot-in-rod light-emitting diodes via surface ligand modification." ACS applied materials & interfaces 10 (2018): 5665-5672.
4. Zeng, Yicheng, et al. "22% Record Efficiency in Nanorod Light-Emitting Diodes Achieved by Gradient Shells." Advanced Materials (2024): 2310705.
5. Mallem, Kumar, et al. "Solution-Processed Red, Green, and Blue Quantum Rod Light-Emitting Diodes." ACS Applied Materials & Interfaces 14 (2022): 18723-18735.
6. Nam, Sooji, et al. "High efficiency and optical anisotropy in double-heterojunction nanorod light-emitting diodes." ACS Nano 9 (2015): 878-885.
7. Zhang, Yongliang, et al. "Highly Efficient Inverted Light-Emitting Diodes Based on Vertically Aligned CdSe/CdS Nanorod Layers Fabricated by Electrophoretic Deposition." ACS Appl. Mater. Interfaces 16, (2024): 10459–10467.
